# Identification of the Sex Pheromone of the Tree Infesting Cossid Moth *Coryphodema tristis* (Lepidoptera: Cossidae)

**DOI:** 10.1371/journal.pone.0118575

**Published:** 2015-03-31

**Authors:** Marc Clement Bouwer, Bernard Slippers, Dawit Degefu, Michael John Wingfield, Simon Lawson, Egmont Richard Rohwer

**Affiliations:** 1 Department of Chemistry/Forestry and Agricultural Biotechnology Institute, University of Pretoria, Pretoria 0002, Gauteng, South Africa; 2 Department of Genetics/Forestry and Agricultural Biotechnology Institute, University of Pretoria, Pretoria 0002, Gauteng, South Africa; 3 Department of Agriculture, Fisheries and Forestry/Ecosciences Precinct, University of the Sunshine Coast, Brisbane, QLD 4001, Australia; 4 Department of Chemistry/Center for Chromatography, University of Pretoria, Pretoria 0002, Gauteng, South Africa; Fundação Oswaldo Cruz, BRAZIL

## Abstract

The cossid moth (*Coryphodema tristis*) has a broad range of native tree hosts in South Africa. The moth recently moved into non-native *Eucalyptus* plantations in South Africa, on which it now causes significant damage. Here we investigate the chemicals involved in pheromone communication between the sexes of this moth in order to better understand its ecology, and with a view to potentially develop management tools for it. In particular, we characterize female gland extracts and headspace samples through coupled gas chromatography electro-antennographic detection (GC-EAD) and two dimensional gas chromatography mass spectrometry (GCxGC-MS). Tentative identities of the potential pheromone compounds were confirmed by comparing both retention time and mass spectra with authentic standards. Two electrophysiologically active pheromone compounds, tetradecyl acetate (14:OAc) and Z9-tetradecenyl acetate (Z9-14:OAc) were identified from pheromone gland extracts, and an additional compound (Z9-14:OH) from headspace samples. We further determined dose response curves for the identified compounds and six other structurally similar compounds that are common to the order Cossidae. Male antennae showed superior sensitivity toward Z9-14:OAc, Z7-tetradecenyl acetate (Z7-14:OAc), E9-tetradecenyl acetate (E9-14:OAc), Z9-tetradecenol (Z9-14:OH) and Z9-tetradecenal (Z9-14:Ald) when compared to female antennae. While we could show electrophysiological responses to single pheromone compounds, behavioral attraction of males was dependent on the synergistic effect of at least two of these compounds. Signal specificity is shown to be gained through pheromone blends. A field trial showed that a significant number of males were caught only in traps baited with a combination of Z9-14:OAc (circa 95% of the ratio) and Z9-14:OH. Addition of 14:OAc to this mixture also improved the number of males caught, although not significantly. This study represents a major step towards developing a useful attractant to be used in management tools for *C. tristis* and contributes to the understanding of chemical communication and biology of this group of insects.

## Introduction

The quince borer *Coryphodema tristis*, Drury, 1782 (Lepidoptera: Cossidae) is native to South Africa. The larvae of this moth has long been known to be a pest of grape vine, apple, quince and sugar pear trees, especially in the Cape Town region [1, 2]. Recently, *C. tristis* was reported from *Eucalyptus nitens* plantations near the Lothair/Carolina area in the Mpumalanga province [3]. Here it causes considerable damage to the trees through extensive tunnelling by the larvae in the wood. *Coryphodema tristis* is, therefore, regarded as a serious emerging threat to the forestry industry in South Africa for which a control programme needs to be developed.

The distribution and population levels of *C. tristis* are difficult to monitor. The greater part of the life cycle, up to eighteen months, is spent as larvae inside the trunks of infested trees [3]. Population levels and the extent of the damage can be confirmed only by felling infested trees, which is obviously not ideal for large-scale assessments. Adult moths are active for a short period in early spring. This narrow period of emergence provides an opportunity to monitor the extent of the infestation indirectly, if a trapping tool were available for the adults.

Sex pheromones are widely used for trapping insects [4]. Lures impregnated with pheromones are typically much more efficient and specific than traps using physical attractants (e.g. light) or with lures imitating chemical signals from the host (kairomones). Pheromones can also be used to disrupt mating by flooding the environment with pheromone to confuse the lured sex [5]. No published studies are available on the pheromone communication of *C. tristis*.

Pheromone attraction between males and females in Lepidoptera is common and well known [6]. Female moths store pheromone molecules or precursors within pheromone glands, that may be dorsally located in the abdomen (for example, the Tiger moth, *Holomelina lamae*, Freeman, 1941), with pores opening in glandular cells between the eighth and ninth abdominal segments [7]. Glandular tubercle structures have been found in other cossid species and possibly function to produce close range pheromones or defense chemicals [8]. Moth pheromones in general are released at specific times to attract a mate. This is occasionally associated with calling behavior such as wing fanning while the ovipositor is exposed to the atmosphere.

Pheromones have been identified for cossid species including the Carpenter moth, *Cossus insularis* [9], the European goat moth, *Cossus cossus* [10] and the Sandthorn carpenter worm, *Holcocerus hippophaecolus* [11]. The identified pheromone components are typically C12–C18 chain length acetates, aldehydes or alcohols with one or two unsaturated positions along the chain. Single compounds either do not attract males [11] or rarely attract as many males in the field as mixtures [10]. This suggests that moths classified in this order generally rely on specific pheromone blends for attraction.

*Coryphodema tristis* males are smaller than females and have plumose antennae, while the females have more simple antennae. This dimorphism in antenna structure suggests that females attract males using pheromones as is typical in the Lepidoptera, including Cossidae. The aim of this study was thus to identify possible pheromone compounds used in sexual communication in *C. tristis*. To achieve this goal, we analyzed female gland extracts and headspace samples through both GC-EAD, GC-MS and GC x GC-MS methods. Electrophysiologically active peaks were identified by both retention times and mass spectra. The relative ratios of the active compounds in the extracts were calculated and dose-response curves measured through GC-EAD for both male and female insects. A field trial was then used to test the biological activity of these compounds.

## Materials and Methods

### Sampling

#### Insects

Infested *Eucalyptus nitens* logs were collected from a plantation near Lothair, South Africa (GPS S 26 18.633, E 30 37.389). The logs were transported to the University of Pretoria and maintained in cages (115 × 65 × 58 cm) in an insectary. This facility had a controlled temperature (20–25°C) and photoperiod (12 hours photo-/scotophase), which was maintained while awaiting emergence of the moths. Male and female moths were separated after emergence.

#### Gland extraction

A total of 62 glands were removed from virgin female moths when calling behavior was observed. Typically, female moths exposed their ovipositors in a series of short intervals while fanning their wings. The glands were removed with clean forceps while gently squeezing the abdomens of the female moths. Twelve glands were extracted in n-hexane and acetone respectively and 38 glands were extracted in dichloromethane. Solvent volumes of a 100 *μ*l were used in each case and glands were extracted for approximately 1 minute to avoid additional contamination that could occur. Extracts that were made for longer periods of time often contained fatty acids with polar carboxylic acid groups. These fatty acids obscured peaks in chromatograms and were not found to be EAD active. Pooling extracts for concentration was attempted but often lead to loss of EAD active peaks and was therefore abandoned.

#### Headspace samples

Headspace samples were collected from individual females overnight. Each female (n = 6) was placed on approximately 0.5 g of silane treated glass wool (Supelco, South Africa) inside a custom made cylindrical glass sampling chamber that was wrapped with aluminum foil to minimize any light disturbances at night. Air was filtered through activated carbon before entering the chamber (13.5 by 5.5 cm, 150 ml internal volume) and passed through the sampling chamber at a flow rate of 47 ± 2.4 ml/min (std). A sampling pump (SKC, South Africa) was used to draw air through the sampling apparatus and through a glass Gerstel thermal desorption sampling trap (17.8 cm x 6 mm x 4 mm ID) containing 60 mm bed length Tenax TA (35 *m* ^2^/*g*). Teflon tubing was used for all connections. Samples were taken from different females for a period of 826 ± 38 min (std) at night. The glass wool inside the sampling chamber was extracted with analytical grade dichloromethane the following morning.

### Electroantennal responses

#### Gas chromatography electro-antennographic detection

GC-EAD recordings were made with an EAD system (Syntech, Hilversum, The Netherlands) coupled to an Agilent 6890N gas chromatography system (Chemetrix, Midrand, South Africa). The antennae from virgin male moths (between 2 and 3 days old) were removed with a surgical blade and the antennal tips were removed in the same manner. This procedure allowed for a superior connection to the tissues inside the antennae and a stable baseline when compared to live insect preparations. The antennae were coupled to two Ag/AgCl capillary glass electrodes filled with an electrolyte solution made by dissolving 3 ml of Spectra®electrolyte gel into 50 ml of distilled water.

The antennal preparation was placed as near as possible (≈ 2 mm) to the outlet of the stimulus delivery tube of the EAD system. Purified and humidified air was allowed to pass through the stimulus delivery tube at a flow rate of 180 ml/min and 2 *μ*l of the gland extract was injected at a split ratio of 5:1 (300°C) onto a 30 m HP 5 analytical column (J & W scientific, 0.32 mm ID, 0.25 *μ*m film). Half of the sample was directed to the antennal preparation through a Y-quartz (Agilent, PN:5181-3398) splitter at the end of the analytical column and the other half to the FID (300°C). The inlet split was used to limit the volumes of solvent to which the antennae were exposed. Direct current recordings were made with a ten times external amplification in all cases and baseline drift was removed by plotting the derivative of the EAD data as described in [12].

The GC was operated in constant pressure mode at 16 psi (He). Two GC oven methods were used. The first oven method (40°C for one minute and ramped to 300°C at 10°C per minute) was used to scan through samples to determine the retention time of electro-physiologically active peaks. The main electro-physiologically active peak eluted at 17.08 minutes when using this method. After the initial EAD screening, the run time was shortened to allow the peak of interest to elute at 9.11 min. The shortened oven method was as follows: 120°C for 1 min and ramped to 220°C at 10°C per minute.

#### Dose response

GC-EAD dose response curves were determined by using the GC (oven 120°C, 1 min—240°C at 20°C/min) to deliver the individual pheromone components at increments of 0.181, 1.81, 18.1, 181 ng/*μ*l. Considering the split ratio of 10:1 in the inlet and the 50% split at the end of the column then 9.09, 90.9, 909, 9090 pg were delivered to the EAD preparation. Live males and females (n = 5) were first wrapped in cotton wool and then in dental wax (Utility wax strips white, Wright Millners) with the head and antennae protruding at the one end. Each insect was coupled to the EAD detector by connecting the reference electrode at the base of the antenna (on top of the main antennal branch near the insect head) and the recording electrode at the tip of the right antenna. This preparation minimized movement of the moths and the decline in antennal sensitivity observed for removed antennae. GC runs were conducted on a 30 m, HP 5 (J & W Scientific, Agilent, 0.32 mm ID, 0.25 *μ*m film) capillary column at 16 psi. The EAD software (GcEad32 V4.3, Syntech, Hilversum, The Netherlands) was used to measure both the FID and EAD response size in terms of the peak height (*μ*V) for the recorded direct current data. The GC was used as a delivery device during these dose response experiments, given the advantages when compared to the normal EAG technique [13].

R version 3.1.0 software package was used to plot dose response curves and to calculate the associated statistical parameters by means of ANCOVA. Dose response parameters were treated as continuous variables and it was assumed that the EAD data should have a log linear relationship with concentration levels [13]. Flame ionization detector data was log(*μ*V+1) transformed to preserve homogeneity among variances of residuals.

### Characterization of chemicals

#### GC-MS

Twenty-seven of the gland extracts that were made in dichloromethane were analyzed on a GC-MS instrument and the remaining samples were analyzed on a GC X GC-MS instrument. The gland extracts were analyzed on a 30 m, HP 5 (J & W Scientific, Agilent, 0.32 mm ID, 0.25 *μ*m film) capillary column with a GC-MS system (HP 5973). The inlet (250°C) was operated in splitless mode (7 psi, He, constant pressure) and 1 *μ*l of the sample was injected (MS solvent delay 2 min). The oven was operated as follows: 40°C for three minutes to 300°C for three minutes at a rate of 20°C/min. Kovats retention indexes were calculated and matched between the GC-EAD instrument and the GC-MS instrument.

#### GC X GC-MS

Samples were injected (250°C) at a 5:1 split ratio onto a GC X GC-TOFMS (Pegasus, Leco) system with a 30 m, ZB 5 (Zebron, 0.25 mm ID, 0.25 *μ*m film) analytical column for the first dimension and a 1 m RXI 17 (Restek, 0.1 ID, 0.1 *μ*m film) column for the second dimension. Helium was used as a carrier gas at 16 psi (constant pressure) for analysis carried out on the ZB 5 capillary column. This analysis was repeated with a 30 m SLB-IL-100 (Supelco, 0.25 mm ID, 0.2 *μ*m film) ionic liquid column to facilitate the separation of the expected Z and E form of the unsaturated component in the gland extract mixture. Here, a 1.59 m ZB 5 column (0.1 *μ*m ID, 0.1 *μ*m film) was used as the second column. A constant flow rate (1 ml/min, He) was used during experiments with the ionic liquid column.

An additional GC x GC-TOFMS (Pegasus, Leco) analysis was done at a split ratio of 10:1 (250°C) with a 30 m SLB-IL-111 column (Supelco, 0.250 mm ID, 0.2 *μ*m film) coupled to a 1.190 m ZB 5 (0.100 mm, 0.1 *μ*m film) as second column. The instrument was operated in constant flow (1 ml/min, He) mode with the primary oven 50–230°C at 10°C/min and the secondary oven 25°C (10°C/min) hotter than primary oven. This analysis was done after the previous analysis for extra confirmation with reference standards. The relative ratios of the compounds in the extracts were calculated by using the two dimensional peak integration results. Standard compounds were obtained in pure form from Insect Science™(Tzaneen, South Africa) and the identities of the peaks were confirmed based on retention times on the ZB5, SLB-IL-100, SLB-IL-111 and HP5 columns.

#### Double bond determination

Dimethyl disulfide (DMDS) adducts of the pheromone extracts (one sample from each solvent used) and standards (1000 ppm in n-hexane) were made according to [14], except that the reaction was carried out at 60°C for 48 hours, not at 40°C overnight. The double bond location of the unsaturated pheromone component in the gland extracts was confirmed by the characteristic ions and retention times when compared to the standard compounds analyzed on the GC X GC-MS system with the SLB-IL 100 and ZB 5 column combination.

### Field trial

The attractiveness of the identified pheromone compounds were assessed in a field trial. The trial was conducted at two sites near the area where original moths had been collected. The trial was undertaken between 27 September and 16 November 2013. Nine treatments were arranged in two stratified random block designs. A pheromone permeation device was constructed with a glass capillary tube (10 mm long, 1.5 mm OD by 0.8 mm ID, ≈ 5 *μ*l internal volume) with one 30 mm long methyl silicone rubber tube (2.16 mm OD by 1.02 mm ID that functioned as a permeation membrane) sealed to both ends of the glass tube so as to form a loop around the latter. Three pheromone compounds (Z9-14:OAc: Z9-14:OH: 14:OAc) or mixtures thereof (≈ 5 *μ*l) were dispensed in each permeation device. Treatments were tested in the following volumetric ratios: treatment one 1: 0: 0; treatment two 0.94: 0: 0.06; treatment three 0.99: 0: 0.01; treatment four 0: 1: 0; treatment five 0.94: 0.06: 0; treatment six 0.06: 0.94: 0; treatment seven 0.95: 0.025: 0.025. Dispensers without pheromone compounds were blank treatments (treatment eight) and newly emerged female insects were used as a positive control (treatment nine). All treatments were replaced twice during the course of the trial (approximately once every 2 weeks). These permeation devices were hung from a wire inside the dispenser area of yellow bucket funnel traps (Insect Science™, Tzaneen, South Africa). Traps were hung at a height of 4 m from standing *E. nitens* trees and they were arranged in a grid pattern with approximately 10 m between traps within the two plantations. Differences between treatments were determined with Steel-Dwass method for non-parametric multiple comparisons.

### Pheromone dispenser release rate

Five replicate permeation dispensers were prepared for Z9-14:OAc, 14:OAc and Z9-14:OH by adding 1 *μ*l of pheromone inside the glass reservoir. Blank dispensers were prepared without pheromone added. The dispensers were kept in an air-conditioned room at 21.5 ± 1.6°C (mean ± std, n = 48). The mass of the dispensers were recorded over a period of 2500 hours with a Mettler Toledo XP6 analytical mass balance with an accuracy of 1 *μ*g. The original mass of the parts of each dispenser was measured before loading with pheromone and was subtracted from each data point. Blank correction was performed by subtracting the mass of the respective pheromone blank dispensers from those containing pheromone. Regression equations and associated statistical parameters for each line fit was determined in R version 3.1.0 by implementing the lm(mass∼time) procedure.

## Results

### Electroantennal responses

#### Gas chromatography electro-antennographic detection

**Fig 1:**
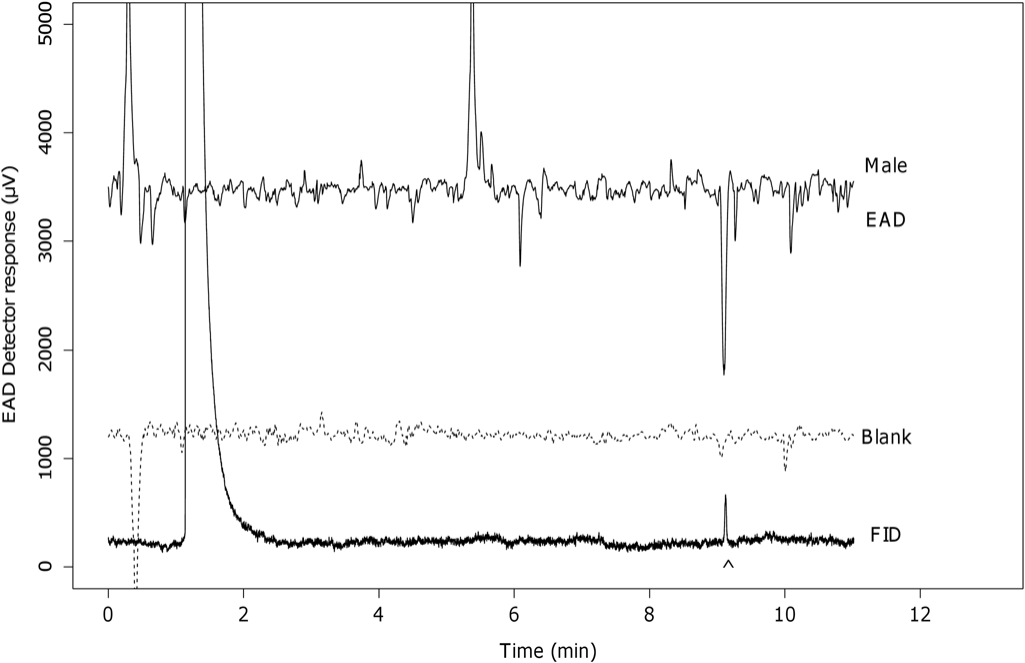
GC-EAD responses of male antennae to gland extracts. The arrow indicates the peak of interest in the FID signal. Bottom is the response to the blank. (EAD response at 9.11 min: 1884 ± 435 *μ*V, mean ± SE, N = 9)

GC-EAD investigation of the gland extracts revealed a large (1.9 ± 0.4 mV, N = 9, ± SE) EAD response of the male antenna to the only peak above the FID detection limit. This provided an estimate of the retention time that could be associated with physiologically relevant peaks in the chromatogram. The response was in most cases well above the noise level of the EAD detector and occurred at 9.11 minutes after optimization of the chromatographic parameters (Fig. 1). The retention index of this peak was calculated as 1797 and matched with the retention index of the standards for Z and E9-14:OAc on this system.

**Fig 2:**
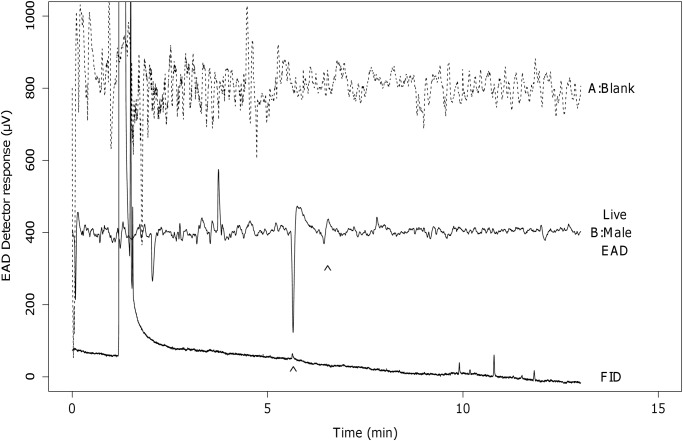
GC-EAD response of male antennae to one of the glass wool extracts of the female headspace. The arrow indicates the peak of interest in the FID signal and the presence of a smaller second response. A: The response to the blank. B: The averaged response of four different sample recordings. (EAD response at 5.61 min: 590 ± 50.33 *μ*V, mean ± SE, N = 4)

Two electroantennographic responses were observed for the glass wool extracts from the female headspace (Fig. 2). The larger response of the two occurred at 5.61 minutes. The retention index of this peak was calculated as 1671.7 on this system. Literature comparison of this retention index suggested that the compound was either E11-14:OH or Z10-14:OH [15] and it was later confirmed to be Z9-14:OH. The smaller response occurred at 6.38 minutes (RI = 1799.0) and coincided with the elution time of Z9-14:OAc on the GC-EAD system. No chromatographic peak could be observed at this response time on both the GC-EAD and GC-MS systems.

#### Dose response

**Fig 3:**
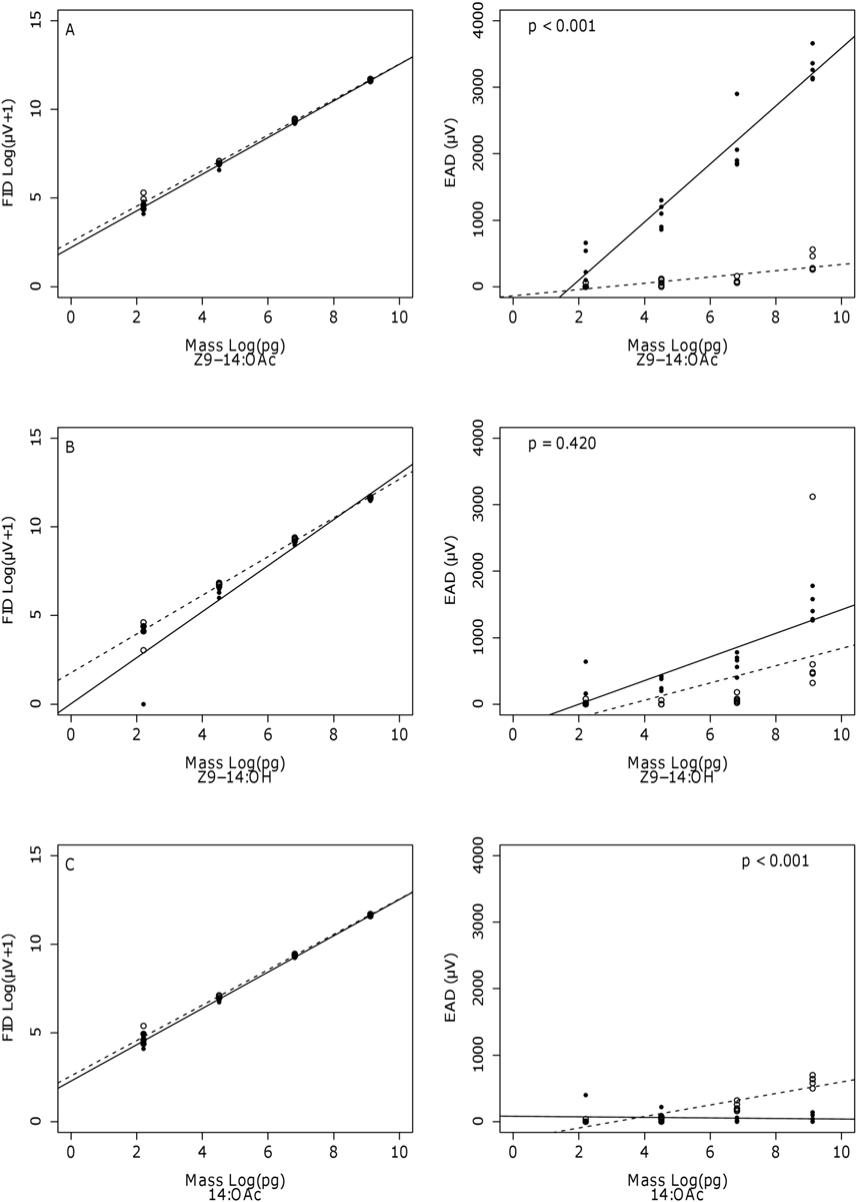
Fitted dose response curves of the FID compared to the EAG response of live *Coryphodema tristis*. Differences between the slope value for the fitted regression lines of males and females are indicated with p values (Ancova) for the EAD data. A = Z9-14:OAc, B = Z9-14:OH, C = 14:OAc. (Dashed lines = Female, Solid lines = Male, N = 5 at each level)

Males and females were treated with similar stimuli for each compound to allow for a direct comparison of the differential sensitivity in the antennae of males and females (Fig. 3 & S1 Fig., S2 Fig.; Table 1, Table 2 & S1 Table, S2 Table). There were five zero values at lower concentrations for Z9-14:Ald, Z9-14:OH and Z11-14:OH. These zero values caused the p value of the interaction term (dose:sex) to become significant only for Z11-14:OH (F = 6.487, p = 0.015). The EAD results confirmed that there was a difference between the sensitivity of male and female antennae. In general males showed larger responses to Z9-14:OAc, Z7-14:OAc, E9-14:OAc and Z9-14:Ald when compared to females. Females were more sensitive only for 14-Ac. Male and female antennae did not show a differential sensitivity for Z11-14:OH and Z9-14:OH. Antennal responses were found to be log linear especially for the male antennae and for those compounds that showed larger responses (for example Z9-14:OAc and Z7-14:OAc).

**Table 1:** Linear fit to dose response curve parameters for FID data for *C. tristis* males and females.

			Intercept (Log(*μ*V+1)				Slope (Log(*μ*V+1)/Log(pg))				Fit parameters	
Compound	Sex	R^2^	Estimate	Std. Error	t value	P value	Estimate	Std. Error	t value	P value	F ratio	P value
Z9-14:OAc	F	0.9954	2.54	0.10	26.27	< 0.001	1.00	0.016	64.46	< 0.001	4155	< 0.001
Z9-14:OH	F	0.9885	1.77	0.17	10.59	< 0.001	1.09	0.027	40.51	< 0.001	1641	< 0.001
14:OAc	F	0.9947	2.58	0.10	24.92	< 0.001	0.99	0.017	59.92	< 0.001	3591	< 0.001
Z9-14:OAc	M	0.9969	2.21	0.08	26.79	< 0.001	1.03	0.013	77.75	< 0.001	6045	< 0.001
Z9-14:OH	M	0.898	0.03	0.62	0.045	0.965	1.30	0.100	12.98	< 0.001	168.4	< 0.001
14:OAc	M	0.9961	2.29	0.09	25.13	< 0.001	1.02	0.015	69.85	< 0.001	4879	< 0.001

*Fit calculations were based on five recordings for each compound at each concentration level

**Table 2:** Linear fit to dose response curve parameters for EAD data for *C. tristis* males and females.

			Intercept (*μ*V)				Slope (*μ*V/log(pg))				Fit parameters	
Compound	Sex	R^2^	Estimate	Std. Error	t value	P value	Estimate	Std. Error	t value	P value	F ratio	P value
Z9-14:OAc	F	0.5998	-135.51	53.93	-2.513	0.022	47.08	8.67	5.429	< 0.001	29.47	< 0.001
Z9-14:OH	F	0.2021	-459.60	335.20	-1.371	0.187	129.90	53.90	2.411	0.027	5.81	0.027
14:OAc	F	0.8224	-267.75	57.04	-4.694	< 0.001	86.51	9.17	9.432	< 0.001	88.97	< 0.001
Z9-14:OAc	M	0.9335	-772.37	165.91	-4.655	< 0.001	436.55	26.68	16.364	< 0.001	267.80	< 0.001
Z9-14:OH	M	0.7570	-355.05	142.45	-2.492	0.023	177.71	22.91	7.758	< 0.001	60.19	< 0.001
14:OAc	M	-0.0122	81.60	54.97	1.484	0.155	-4.17	8.84	-0.472	0.643	0.22	0.6428

*Fit calculations were based on five recordings for each compound at each concentration level

### Characterization of chemicals

#### GC-MS

A large peak was found at elution time of 11.802 minutes during the GC-MS investigation with a smaller peak eluting at 11.857 minutes (S3 Table). The calculated retention indexes compared well with the literature values [15] for both Z9-14:OAc and/or E9-14:OAc and 14:OAc for the smaller peak (Table 3). The library search indicated that the first peak was either Z and/or E9-14:OAc and it was possible that they may have co-eluted on the DB 5 column [15]. The second peak was tentatively identified as 14:OAc and this compound and Z9-14:OAc was later confirmed with the reference standards. Z9-14:OAc was the dominant component in the gland extracts of females and was present in a relative ratio of 94.4 ± 3.8%: 5.6 ± 3.7% (mean ± SD, N = 27) when compared to 14:OAc.

**Table 3:** Kovats retention index comparison of standard compounds between the GC-MS and GC-EAD (HP5, 0.32 mm, 0.25 *μ*m).

Compound	Purity	RI GC-MS	RI GC-EAD
Z9-14:Ald	95%	1604.7	1605.6
Z9-14:OH*	98%	1666.1	1665.4
Z11-14:OH*	95%	1678.4	1676.1
Z5-14:OAc	97%	1789.9	1788.5
Z7-14:OAc	97%	1792.0	1790.5
Z9-14:OAc	97%	1799.8	1798.7
E9-14:OAc	96%	1800.4	1798.5
14:OAc	98%	1809.5	1808.7
Z11-14:OAc	95%	1811.7	1809.7

* peak start time used to calculate RI

GC-MS, oven 40, 3 min to 300 @ 20°C/min, 3 min, 7 psi, He, 48.9 cm/sec, Butane 80°C

GC-EAD, oven 120, 1 min to 300 @ 20°C/min, 3 min, 16 psi, He, 47.1 cm/sec, Butane 80°C

Mass spectral comparison of the peak found in the headspace with the NIST library indicated that the compound was either Z9-14:OH or Z or E11-14:OH. This peak had the same retention index of 1667 (Table 4) as was reported for Z9-14:OH [15]. Using a reference standard, this peak was later confirmed to be Z9-14:OH.

**Table 4:** Retention time and Kovats retention index of active compounds found in *C. tristis* headspace samples.

	Headspace		Glass wool	
Sample	RT (min)	RI	Rt (min)	RI
1	12.652	1667.0	11.162	1667.2
2	12.630	1666.1	11.162	1667.2
3	12.603	1667.2	11.170	1667.3
4	12.614	1669.1	*	*

* Not detected

#### GC X GC-MS

Separation of the compounds present in the gland extracts was achieved with the polar column (SLB-IL-111) used in the GC x GC-MS instrument. The mass spectrum and retention times of the unknown peaks were compared with those obtained for the standard compounds and the library hit spectrum (S4 Table). The characteristic ions (m/e = 348 (M^+^), 231, 117) of the DMDS adducts revealed the double bond location between the ninth and tenth carbon atoms of the unsaturated C14 acetate (S3 Fig.). The geometry around the double bond was confirmed to be Z and not E as compared to retention time differences between derivatised individual standards and samples.

The ratio of the compounds in the gland extracts was found to be slightly different when samples were analyzed on the two-dimensional instrument. This instrument could separate E9-14:OAc and Z9-14:OAc and the results showed that E9-14:OAc could not be detected in any of these extracts (S4 Table). The fact that E9-14:OAc was not detected in these extracts, suggests that it is not part of the pheromone blend of *C. tristis*. These analyses revealed a ratio of 1.13 ± 1.31: 98.87 ± 1.31 (14:OAc: Z9-14:OAc, mean ± SD, N = 33). Two of the dichloromethane extracts did not contain the compounds above the detection limit and were thus not included in the calculation of the ratio.

### Field trial

Results of the field trial showed that the attractiveness of some of the artificially baited traps was enhanced, if compared to blank traps. A total of 122 male moths were caught in the field trial and 102 of these were caught using treatment 5 and 7, which contained Z9-14:OAc, Z9-14:OH (94: 6%) and Z9-14:OAc, Z9-14:OH, 14:OAc (95: 2.5: 2.5%) respectively. A greater number of males were caught when all three components; Z9-14:OAc, Z9-14:OH, 14:OAc, identified from the gland and headspace samples were used in the pheromone lures (treatment no 7), although this did not differ significantly (p = 0.6667, Steel-Dwass) from treatment 5 (Table 5). No moths were caught in blank treatments and only two of 30 female *C. tristis* moths that were used as positive control lured a small number (8) of male moths. The time period that female *C. tristis* moths remained alive within the traps was unknown because all females were found to be dead upon treatment replacement. This possibly contributed to the low numbers that were caught by the females.

**Table 5:** Field trial treatment ratios, by volume, and number of males caught per treatment.

n	Treatment	Z9-14:OAc	Z9-14:OH	14:OAc	# Males	Letters*
10	1	1	0	0	2	AB
10	2	0.94	0	0.06	6	AB
10	3	0.99	0	0.01	3	AB
10	4	0	1	0	0	A
10	5	0.94	0.06	0	39	BC
10	6	0.06	0.94	0	1	A
10	7	0.95	0.025	0.025	63	BC
10	8 (Blank)	0	0	0	0	A
10	9 (Female)	0	0	0	8	AB

*Rows with the same letters are not statistically significantly different Steel-Dwass, p < 0.05

### Pheromone dispenser release rate

All dispensers showed a steady loss of mass before blank correction. This mass loss was partially due to degradation of the permeation disperser itself, evidenced by the mass loss observed for the blank dataset. After blank correction mass loss was evident only for Z9-14:OAc (-62.5 ± 9.0 ng/hour, mean ± SE) and 14:OAc (-49.7 ± 16.0 ng/hour, mean ± SE), whereas the alcohol, Z9-14Ol (+38.7 ± 18.9 ng/hour, mean ± SE) showed a steady mass gain (S4 Fig.). It was likely that the hydrophilic nature of the alcohol compound caused water absorption from the atmosphere.

## Discussion

Analysis of pheromone gland extracts from the cossid moth, *C. tristis* in this study revealed that Z9-14:OAc and 14:OAc were possible pheromone candidates. Headspace samples contained the additional compound Z9-14:OH. All three of these compounds were detected by male antennae. Pheromone lures containing at least Z9-14:OAc and Z9-14:OH in a specific ratio significantly increased the number of males caught in the field trials, confirming the biological activity of these two compounds. The addition of 14:OAc to lures also increased the number of moths trapped, although this difference was not statistically significantly different from lures containing Z9-14:OAc and Z9-14:OH. These results represent a major step towards developing an environmentally friendly monitoring and management tool for *C. tristis* in South Africa.

Behavioral data were difficult to collect for *C. tristis* under laboratory conditions (data not shown). Adults of this species are available only for a very short period (approximately 1.5 months) each year. Females reared from field-collected infested logs were often observed to have a few males in close proximity (often less than 10 cm) inside cages, but despite intensive monitoring, mating was never observed. Delaying the emergence period through lowering the ambient temperature of logs was attempted, but this often resulted in fungal growth over the logs and only a few stunted moths that emerged. These observations suggest that the change in environmental conditions, such as lack of some environmental cues or other changes brought about when moths are reared in captivity, may have a direct influence on the mating behavior of *C. tristis*.

The dose response experiments indicated that the double bond position, its geometry and the functional group play an important role in the recognition system of the male *C. tristis*. In general, the male antennal response size became larger when the double bond position was moved to the seventh and ninth carbon positions. Their antennae were also more sensitive to the acetate functionality and the Z geometry around the double bond (see for example the responses to Z9-14:OAc compared to E9-14:OAc and Z9-14:OH). These results confirmed that the pheromone receptors present on the male antennae are selectively sensitive to Z9-14:OAc, Z7-14:OAc, E9-14:OAc and Z9-14:OH when compared to females. Similar electrophysiological patterns are known for other moths residing in this family of the Lepidoptera. For example, European goat moth (*Cossus cossus*) males show greater antennal responses towards C12 acetates with the (Z)-geometry at the double bond at the fifth carbon as compared C12 alcohols [10]; and males of the Sandthorn Carpenterworm (*Holcocerus hippophaecolus*) also show larger antennal responses to Z7-14:OAc when compared to the corresponding alcohol [11]. The compounds that elicit larger antennal responses were also the main pheromone components for these species.

We expected the antennal response to have a log linear relationship to stimulus concentration at levels below saturation [13, 16]. This was indeed the case for male responses to Z9-14:OAc and Z7-14:OAc and both male and female responses to Z11-14:OAc. This result suggests that the tested pheromone concentration was below the saturation level for at least these compounds.

Pheromone concentration plays an important role in the selectivity process of the antennae. For example, Mayer [17] could show that three different pheromone molecules (for *Trichoplusia ni*) stimulate three different receptor neurons at physiologically relevant concentration levels, but all three neurons were sensitive to all three compounds at concentrations above physiological levels. Our results indicate that female antennae saturate rapidly when compared to males especially for Z9-14:OAc, E9-14:OAc and Z7-14:OAc. Males should, therefore, have greater numbers of receptors for these compounds on their antennae when compared to females, which possibly relates to the larger surface area of their antennae.

Female moth antennae are in most cases expected to be less sensitive to their own pheromone molecules (for example the silk worm moth *Bombix mori*, [18] and the turnip moth, *Agrotis segetum*, [19], but exceptions of pheromone auto-detection in females do occur [20]. Our dose response experiments showed that males are more sensitive than females to most of the tested pheromone compounds. The results show that there is indeed an auto-detection that occurs for *C. tristis* females, but this was detected only at the higher concentrations tested.

Pheromone molecules similar to those that were identified here have been found to be components of pheromones in other moth species residing in the sub-family Cossinae. These include the European goat moth, *Cossus cossus* [10] and the sandthorn carpenterworm, *Holcocerus hippophaecolus* [11, 21]. The similarity of pheromone compounds utilized in Cossinae, including *C.tristis* indicate that these molecules are relatively common and that specificity lies in unique combinations. For example, Fang et al., [11] found that the individual compounds Z7-14:OAc and E3-14:OAc failed to attract males in the field. However, mixing them in a 1:1 ratio restored their attraction. The results of the present study show that *C. tristis* males also rely on such a synergistic ratio for attraction to occur. Attraction is enhanced when both the acetate (Z9-14:OAc) and alcohol (Z9-14:OH) are present within the artificial lures. The function of 14:OAc in the pheromone blend is unknown at this stage, but traps containing a small fraction of this compound caught a greater number of male moths.

This study revealed the identity of three electro-physiologically and behaviorally active compounds in the cossid moth *C. tristis*, two of which were found in the female gland extracts, Z9-14:OAc and 14:OAc, and another in the headspace, Z9-14:OH. It is possible that some components, Z9-14:OAc and 14:OAc, of the pheromone are stored within the gland and another, Z9-14:OH, is produced at the time of calling. Blends as opposed to single compounds were more effective for luring male moths into traps in the field. Future work should focus on enhancing trap efficiency by optimizing the lure release rates to match that of a calling female moth. It is also possible that additional undiscovered minor components could be present in the pheromone plumes of calling females. These compounds were below detection capabilities of the present study, but they may yet be discovered.

## Supporting Information

S1 Fig Fitted dose response curves of the FID compared to the EAG response of live *Coryphodema tristis*. D = E9-14:OAc, E = Z11-14:OAc and F = Z11-14:OH.
(TIF)

S2 Fig Fitted dose response curves of the FID compared to the EAG response of live *Coryphodema tristis*. G = Z5-14:OAc, H = Z7-14:OAc and I = Z9-14:Ald.
(TIF)

S3 Fig A comparison between mass spectra of the suspected pheromone compounds. A: The mass spectrum found for a hexane gland extract at the region of interest. B: The mass spectrum of the standard compound Z9-14:OAc. C: The library comparison to the gland sample. D: The mass spectrum of the DMDS adduct from a sample in n-hexane.
(TIF)

S4 Fig Gravimetric pheromone release rate determination of pheromone permeation devices. Regression line slope values were used to estimate pheromone release rate.
(TIF)

S1 Table Linear fit to dose response curve parameters for FID data for *C. tristis* males and females. (TIF)

S2 Table Linear fit to dose response curve parameters for EAD data for *C. tristis* males and females. (TIF)

S3 Table Chromatographic peak data of pheromone gland extract samples analyzed on the GC-MS. (TIF)

S4 Table Chromatographic peak data of pheromone gland extract samples analyzed on the GC*GC-MS. (TIF)

## References

[1] Petty, F. The quince borer and its control. 1917; Bulletin No 2 Department of Agriculture.

[2] Meyer AJ. A histological study of the alimentary canal and associated structures in the larva of *Coryphodema tristis* Drury (Lepidoptera). 1965; Ph.D. thesis, Stellenbosch: Stellenbosch University.

[3] Gebeyehu S, Hurley BP, Wingfield MJ. A new lepidopteran insect pest discovered on commercially grown *Eucalyptus nitens* in South Africa: research in action. South African Journal of Science. 2005;101: p-26.

[4] Hegazi E, Khafagi W, Konstantopoulou M, Raptopoulos D, Tawfik H, Abd El-Aziz G, et al Efficient mass-trapping method as an alternative tactic for suppressing populations of leopard moth (Lepidoptera: Cossidae). Annals of the Entomological Society of America. 2009;102: 809–818. 10.1603/008.102.0507

[5] Carde RT, Minks AK. Control of moth pests by mating disruption: successes and constraints. Annual review of entomology. 1995;40: 559–585. 10.1146/annurev.en.40.010195.003015

[6] Jurenka R. Insect pheromone biochemistry and molecular biology, Oxford: Elsevier Academic Press, chapter Biochemistry of female moth sex pheromones 2003; pp. 53–80.

[7] Ma PW, Ramaswamy SB. Insect pheromone biochemistry and molecular biology, Oxford: Elsevier Academic Press, chapter Biology and ultrastructure of sex pheromone-producing tissue 2003; pp. 19–51.

[8] Davis SR. Description of a new lepidopteran structure, the abdominal tubercles. Journal-Lepidopterists Society. 2006;60: 194.

[9] Chen X, Nakamuta K, Nakanishi T, Nakashima T, Tokoro M, Mochizuki F, et al Female sex pheromone of a carpenter moth, *Cossus insularis* (Lepidoptera: Cossidae). Journal of Chemical Ecology. 2006;32: 669–679. 10.1007/s10886-005-9025-4 16683203

[10] Capizzi A, Tonini C, Arsura E, Guglielmetti G, Massardo P, Piccardi P. Sex pheromone components of the European goat moth, *Cossus cossus* . Journal of Chemical Ecology. 1983;9: 191–200. 10.1007/BF00988036 24407337

[11] Fang YL, Sun JH, Zhao CH, Zhang ZN. Sex pheromone components of the sandthorn carpenterworm, *Holcocerus hippophaecolus* . Journal of Chemical Ecology. 2005;31: 39–48. 10.1007/s10886-005-0972-6 15839478

[12] Slone D, Sullivan B. An automated approach to detecting signals in electroantennogram data. Journal of Chemical Ecology. 2007;33: 1748–1762. 10.1007/s10886-007-9338-6 17668268

[13] Struble DL, Arn H. Combined gas chromatography and electroantennogram recording of insect olfactory responses In: Techniques in Pheromone Research, Springer 1984; pp. 161–178. 10.1007/978-1-4612-5220-7_6

[14] Buser HR, Arn H, Guerin P, Rauscher S. Determination of double bond position in mono-unsaturated acetates by mass spectrometry of dimethyl disulfide adducts. Analytical Chemistry. 1983;55: 818–822. 10.1021/ac00257a003

[15] Marques FdA, McElfresh J, Millar JG. Kováts retention indexes of monounsaturated c12, c14, and c16 alcohols, acetates and aldehydes commonly found in lepidopteran pheromone blends. Journal of the Brazilian Chemical Society. 2000;11: 592–599. 10.1590/S0103-50532000000600007

[16] Mayer M, Mankin R, Lemire G. Quantitation of the insect electroantennogram: measurement of sensillar contributions, elimination of background potentials, and relationship to olfactory sensation. Journal of Insect Physiology. 1984;30: 757–763. 10.1016/0022-1910(84)90041-6

[17] Mayer M. Responses of three antennal specialist neurons of male *Trichoplusia ni* (Hübner) to sex pheromone components at and above naturally emitted levels. Journal of Insect Physiology. 1993;39: 401–412. 10.1016/0022-1910(93)90028-P

[18] Schneider D. Elektrophysiologische untersuchungen von chemo-und mechanorezeptoren der antenne des seidenspinners *Bombyx mori* l. Zeitschrift für vergleichende Physiologie. 1957;40: 8–41. 10.1007/BF00298148

[19] Hansson BS, Pers JNVD, Löfqvist J. Comparison of male and female olfactory cell response to pheromone compounds and plant volatiles in the turnip moth, *Agrotis segetum* . Physiological Entomology. 1989;14: 147–155. 10.1111/j.1365-3032.1989.tb00946.x

[20] Schneider D, Schulz S, Priesner E, Ziesmann J, Francke W. Autodetection and chemistry of female and male pheromone in both sexes of the tiger moth *Panaxia quadripunctaria* . Journal of Comparative Physiology A. 1998;182: 153–161. 10.1007/s003590050166

[21] Fang YL, Sun JH, Zhao CH, Yong-Ping S, Hong-Qi Z, et al Identification of sex pheromone components of *Holcocerus hippophaecolus* (Lepidoptera: Cossidae) and their biological activities. Acta Entomologica Sinica. 2003;5.

