# Supplementary figures and images for: Identification of the Sex Pheromone of the Tree Infesting Cossid Moth Coryphodema tristis (Lepidoptera: Cossidae)
Source: PLoS One. 2015 Mar 31;10(3):e0118575. doi: 10.1371/journal.pone.0118575 (PMC4380472; doi:10.1371/journal.pone.0118575)

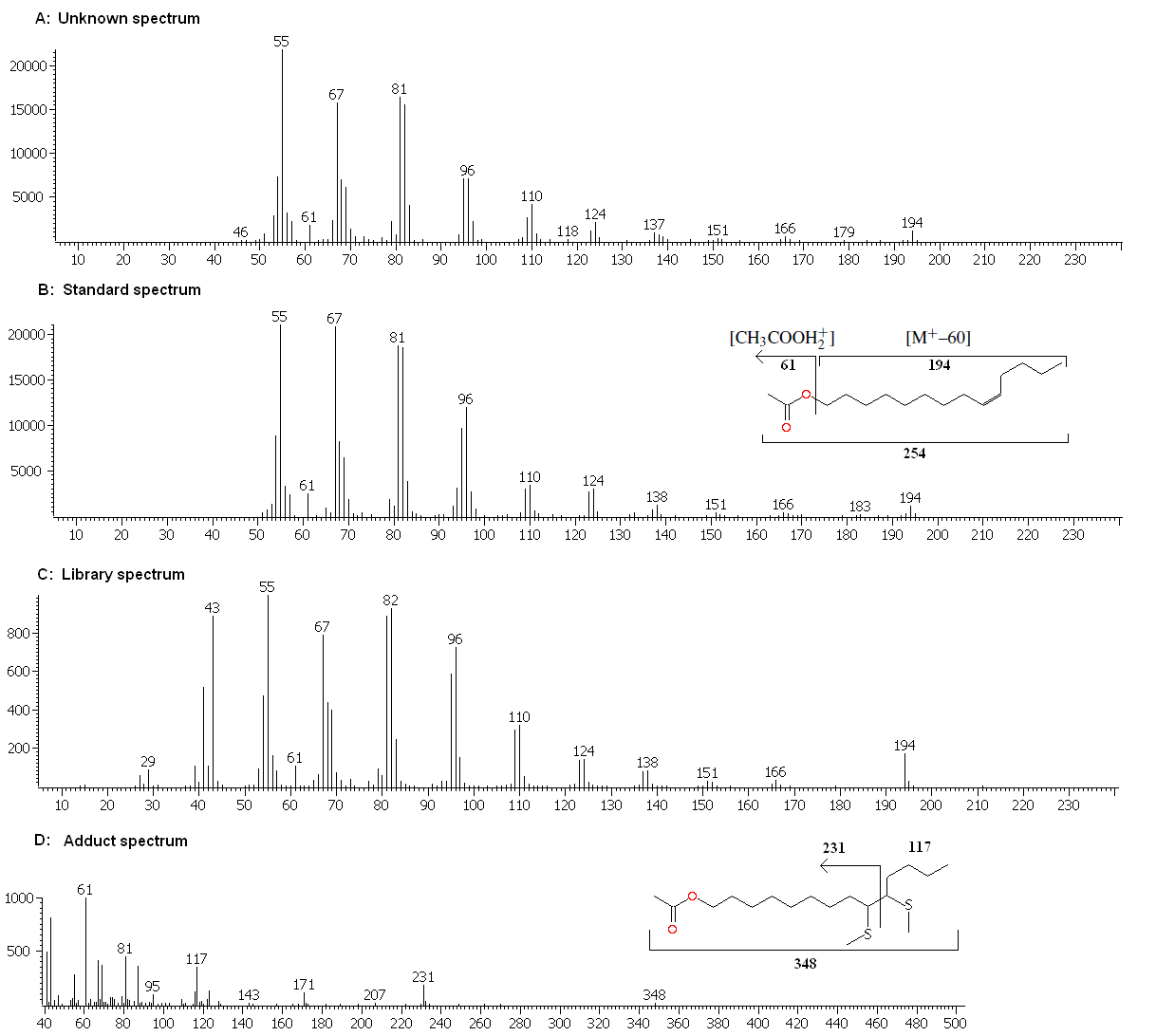

Supplement: S1 Fig — D = E9-14:OAc, E = Z11-14:OAc and F = Z11-14:OH. (TIF) [file pone.0118575.s001.tif]

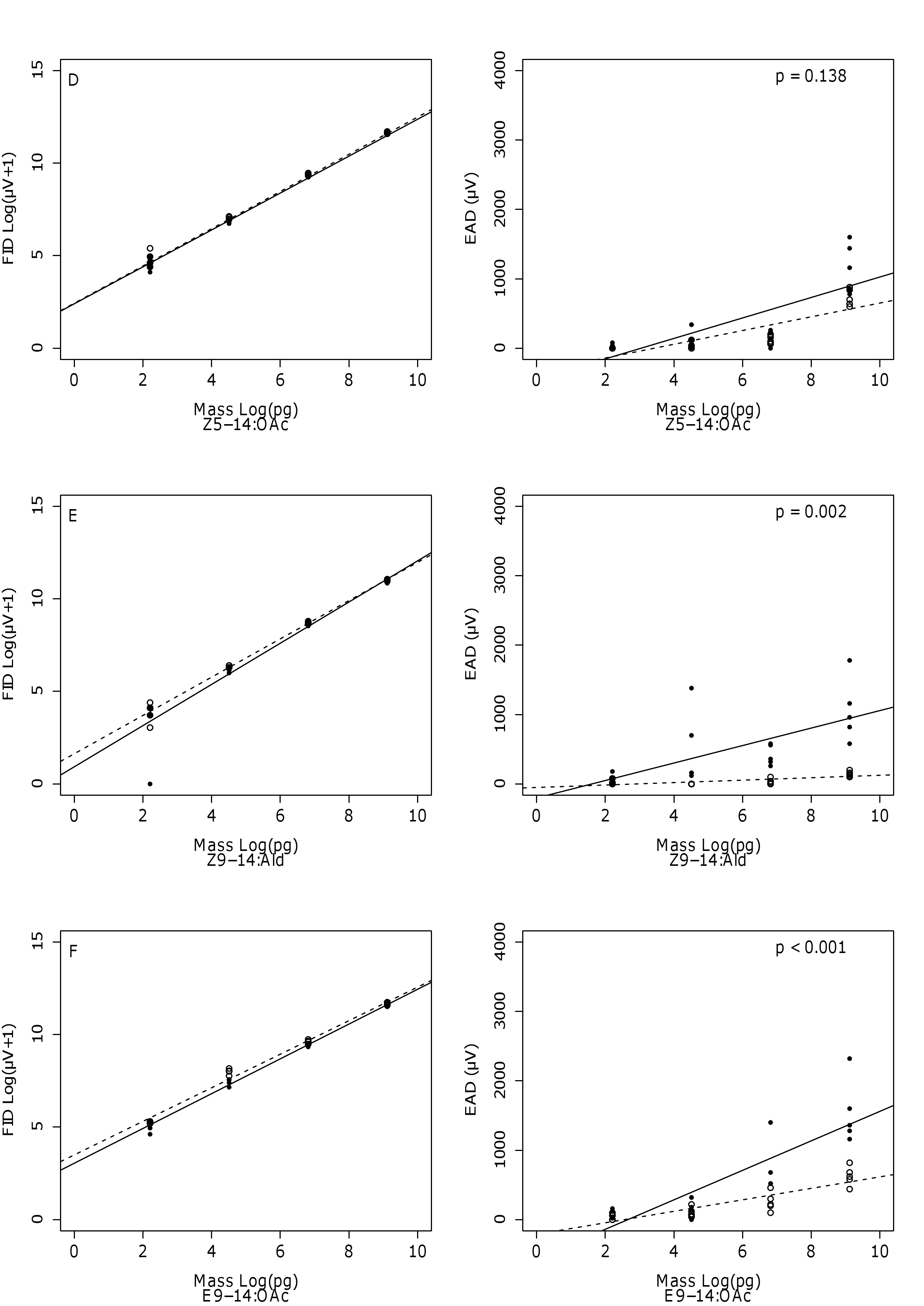

Supplement: S2 Fig — G = Z5-14:OAc, H = Z7-14:OAc and I = Z9-14:Ald. (TIF) [file pone.0118575.s002.tif]

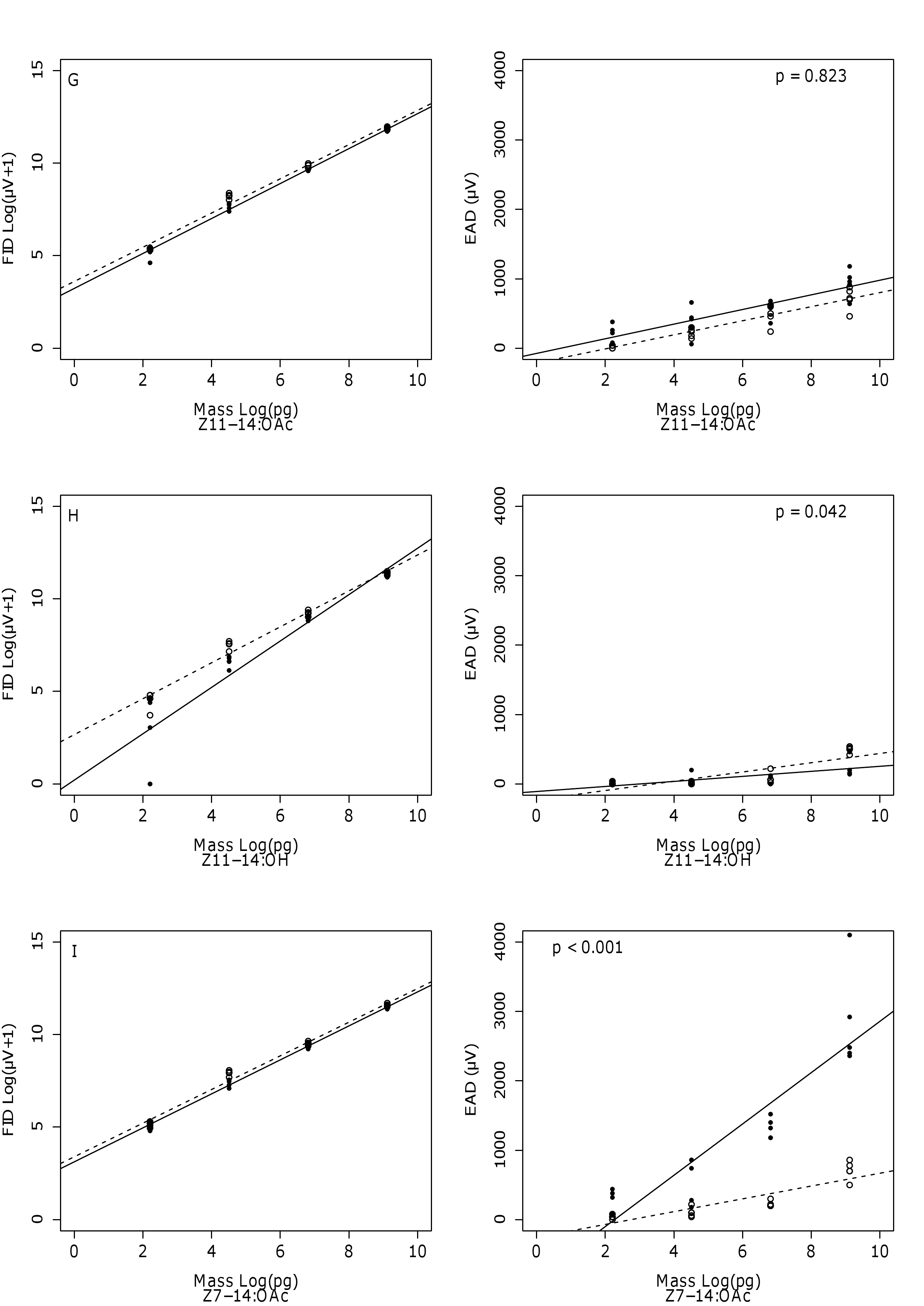

Supplement: S3 Fig — A: The mass spectrum found for a hexane gland extract at the region of interest. B: The mass spectrum of the standard compound Z9-14:OAc. C: The library comparison to the gland sample. D: The mass spectrum of the DMDS adduct from a sample in n-hexane. (TIF) [file pone.0118575.s003.tif]

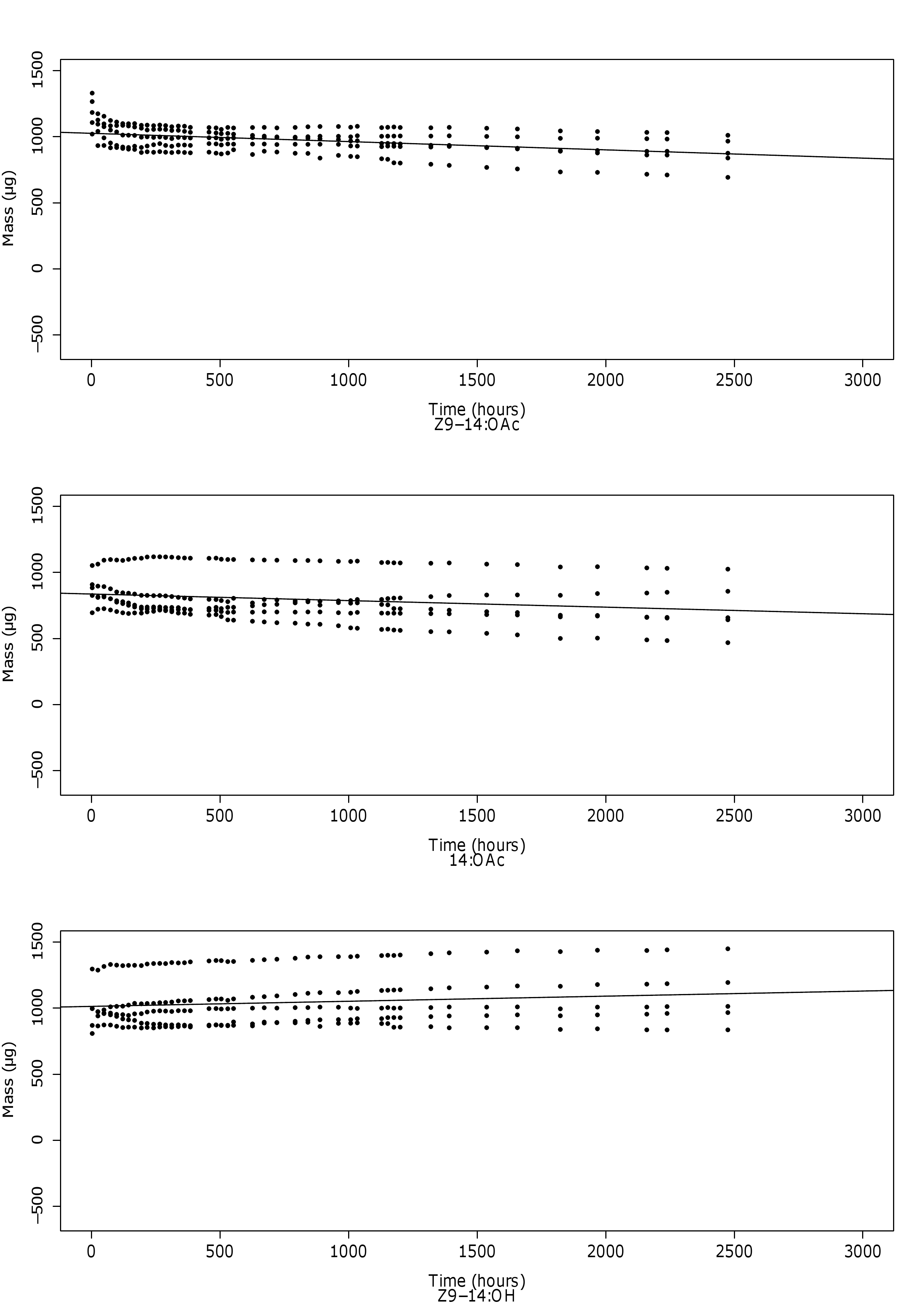

Supplement: S4 Fig — Regression line slope values were used to estimate pheromone release rate. (TIF) [file pone.0118575.s004.tif]

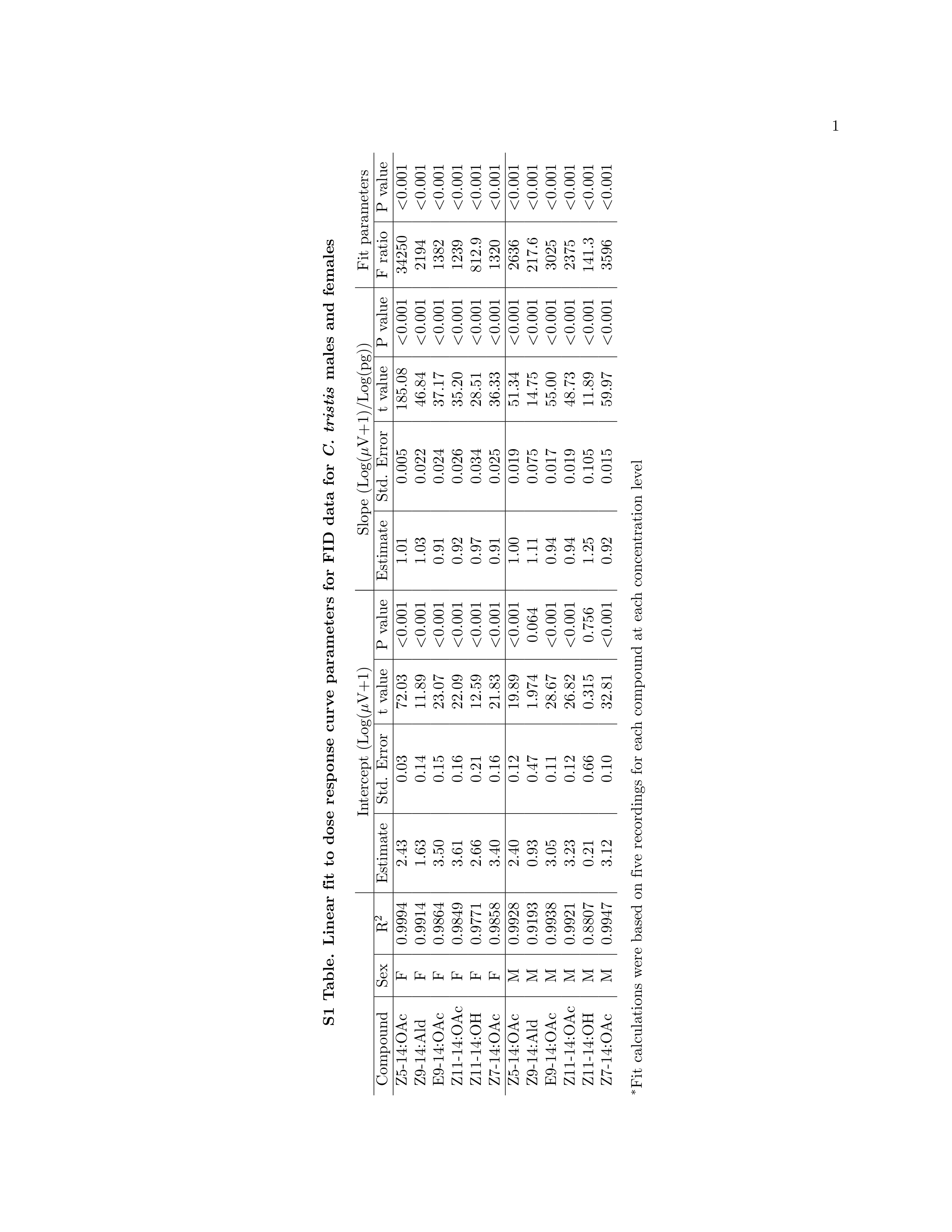

Supplement: S1 Table — (TIF) [file pone.0118575.s005.tif]

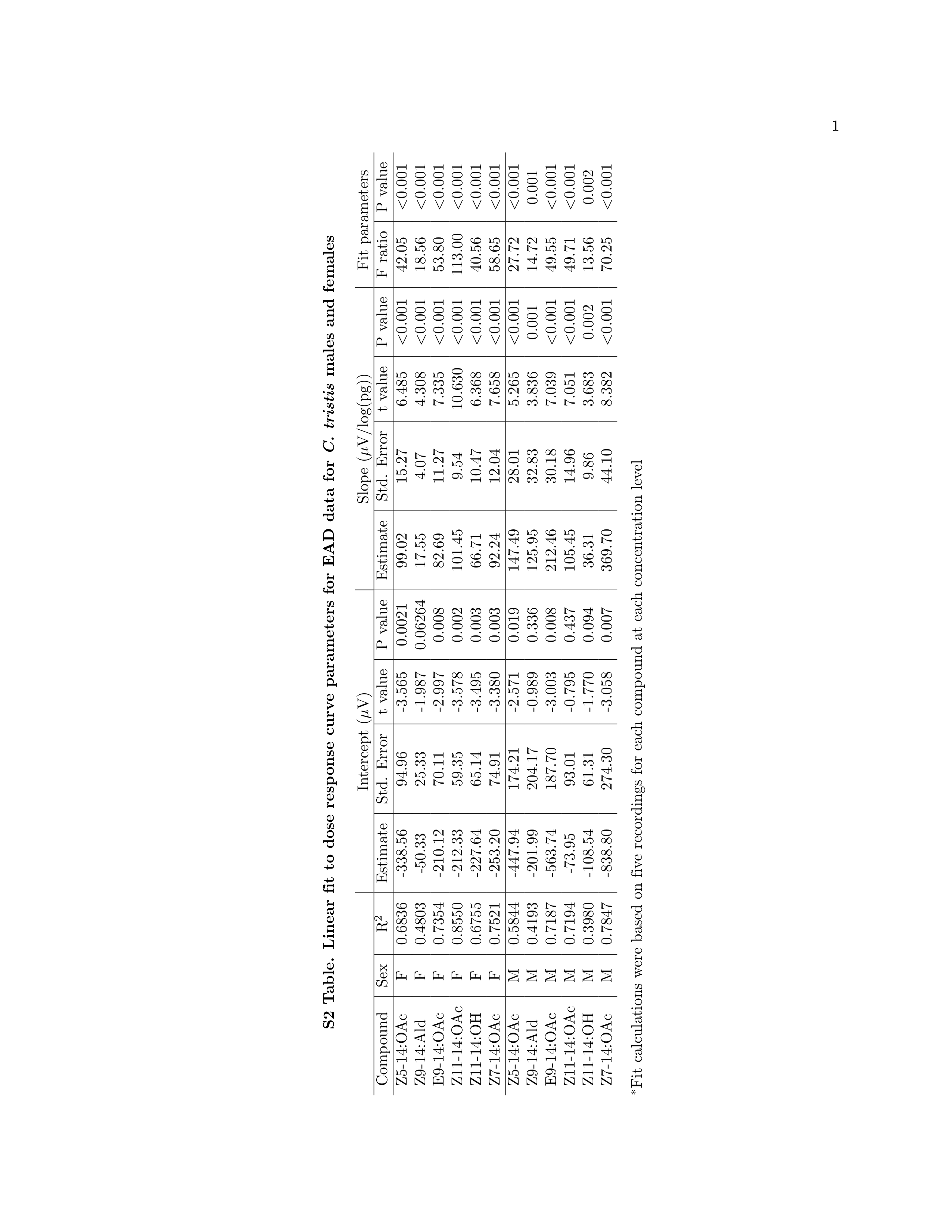

Supplement: S2 Table — (TIF) [file pone.0118575.s006.tif]

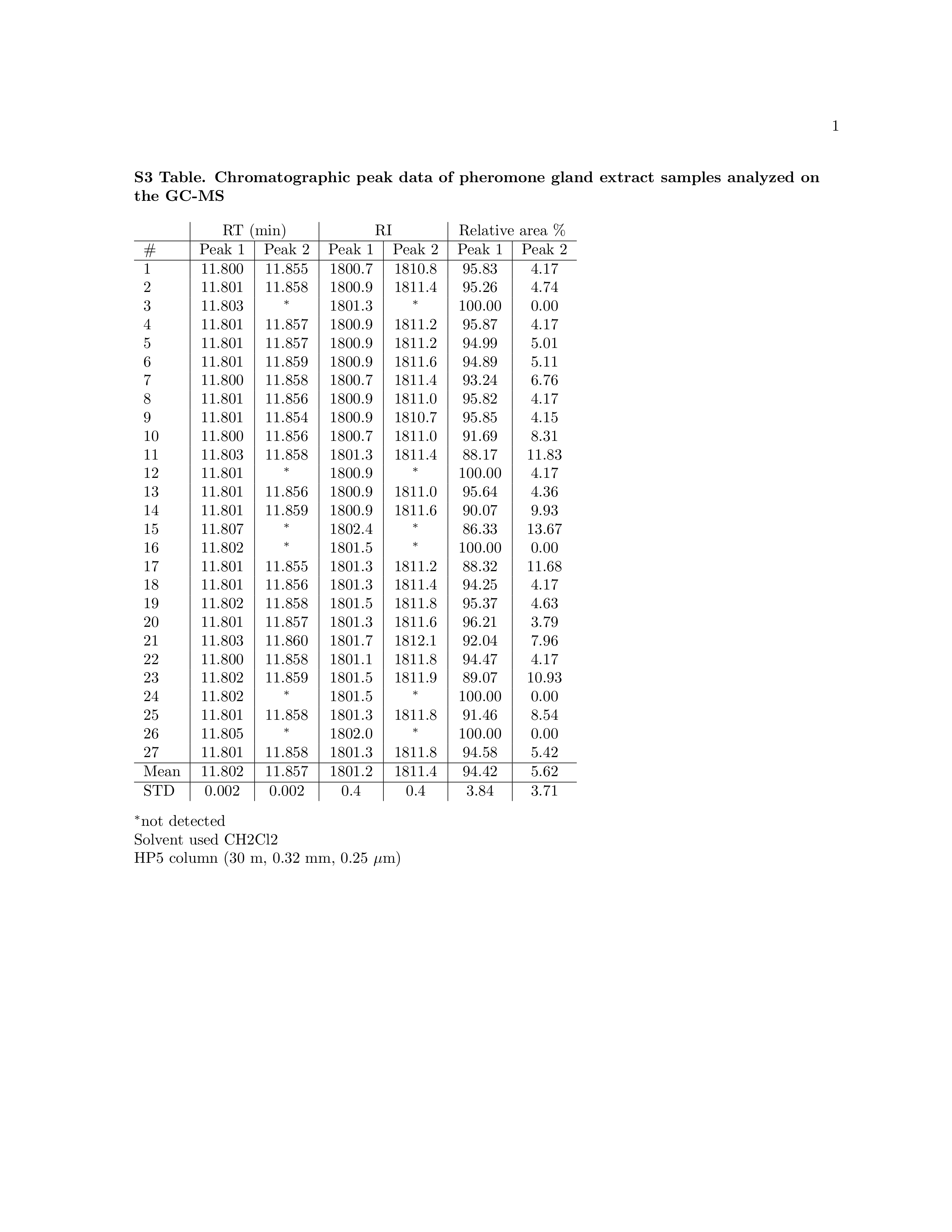

Supplement: S3 Table — (TIF) [file pone.0118575.s007.tif]

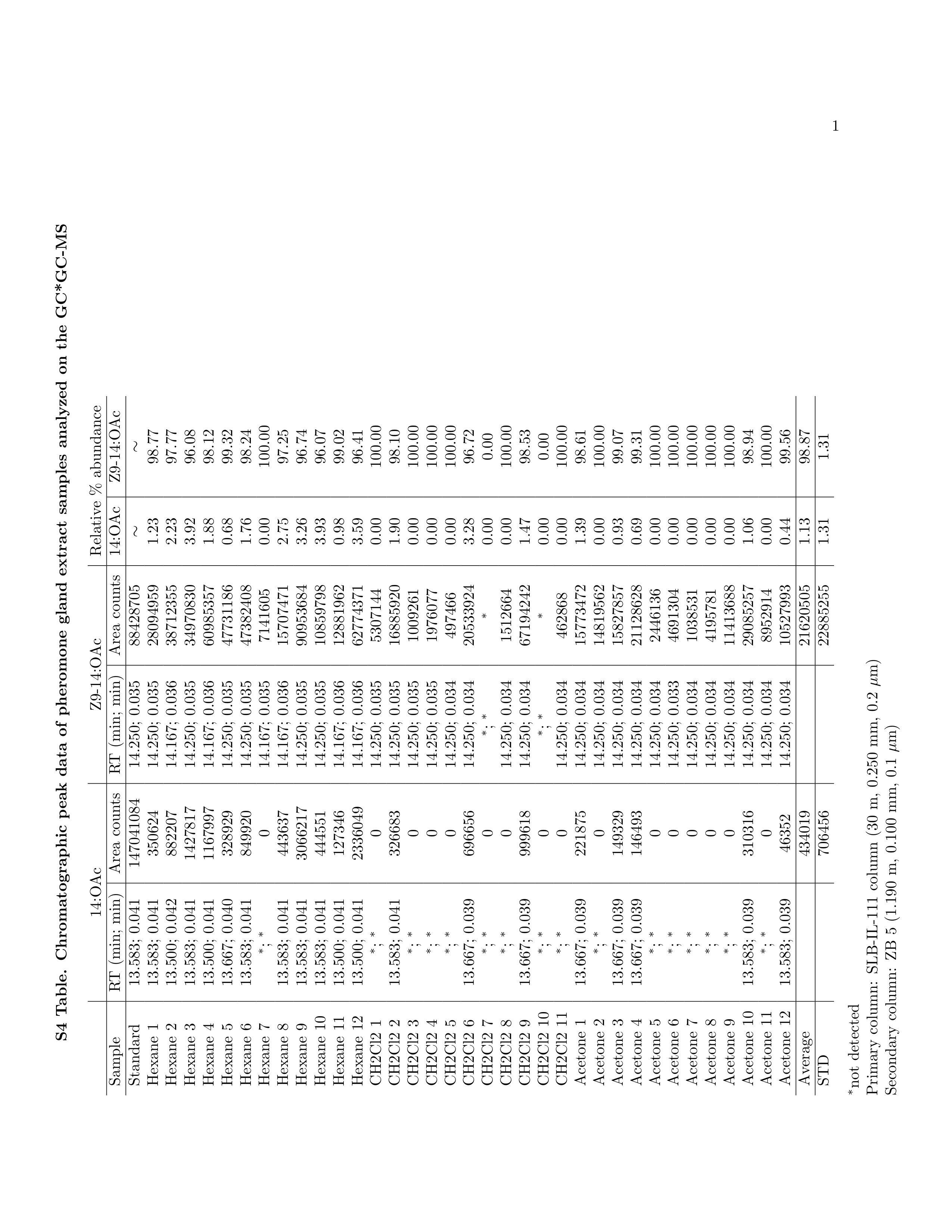

Supplement: S4 Table — (TIF) [file pone.0118575.s008.tif]
